# Against all odds—the persistent popularity of homeopathy

**DOI:** 10.1007/s00508-020-01624-x

**Published:** 2020-03-09

**Authors:** Cemre Cukaci, Michael Freissmuth, Christopher Mann, Joshua Marti, Veronika Sperl

**Affiliations:** 1 Association of Medical Students, Medical University of Vienna, Währinger Gürtel 18–20, 1090 Vienna, Austria; 2 Institute of Pharmacology, Centre of Physiology and Pharmacology, Medical University of Vienna, Währinger Str. 13a, 1090 Vienna, Austria

**Keywords:** Evidence-based medicine, Clinical trials, Homeopathy, Placebo, Responsible patient

## Abstract

The use of homeopathy is remarkably popular. Popularity, however, is not an arbiter in a scientific discourse. In fact, the assumptions underlying homeopathy violate fundamental laws of nature. Homeopathy does not have any explanatory power and fails other criteria established for a scientific approach. Two large-scale efforts have recently documented that in spite of a plethora of clinical trials there is no evidence that homeopathic remedies have any therapeutic effect, which goes beyond that of a placebo. Relaxed regulations and lack of scientific literacy and of health education allow for continuous thriving of homeopathy. While the tide may be changing on the regulatory side, health education of the general public is presumably more important to support informed decision making by patients. Otherwise, the responsible patient, who is posited to decide on the medical choices, remains a convenient legal fiction.

## Introduction

Homeopathy is a remarkably successful system of beliefs. Its success can be gauged from the fact that homeopathic remedies are exempt from most regulatory requirements, which are otherwise imposed on marketed drugs. These exemptions pertain to adherence to good manufacturing practice (GMP) standards, to demonstration of safety of efficacy and to pharmacovigilance (see e.g. Directive 2001/83/EC of the European Union and ref. [1]). Proponents of homeopathy argue that the use of homeopathy has been stable over time (1986–2012) and that it is used on a worldwide basis [2]. In fact, the historical record suggests otherwise: homeopathy was more prevalent in the early twentieth century than it is nowadays. There were, for instance, 20 medical homeopathic colleges in the USA in the early 1900s, but over the next 50 years they all disappeared [3]. Similarly, homeopathy rose to prominence in interwar Germany because of the purported “crisis” of academic medicine [4], which was perceived as soulless and materialistic. Importantly, ideological foes, i.e. Jews and Freemasons, supposedly had a stranglehold over academic medicine (“verjudete und verfreimaurte Schulmedizin”). Homeopathy was part of the recovery program to establish the *Neue Deutsche Heilkunde* (New German Medicine), which would support the supremacy of the Aryan race [5]. Homeopathy and other forms of “alternative” or “complementary” medicine saw a revival in the late twentieth century [6]. This period also saw the emergence and continuous refinement of evidence-based medicine: at present, guidelines are provided in essentially all fields and subdisciplines; these guidelines meticulously document the level of evidence and the resulting grade of recommendation for both diagnostic approaches and interventions. In addition, they are revised on a regular basis to account for progress and new insights from (large) clinical trials. The popularity of homeopathy and other forms of “alternative/complementary” medicine is in stark contrast to the dearth of evidence to support its efficacy.

**Fig. 1:**
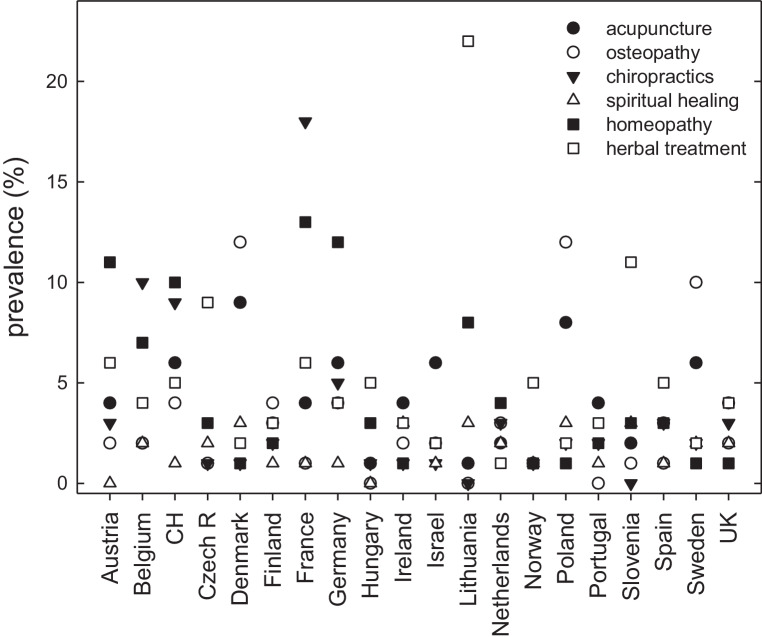
Prevalence of “complementary/alternative” medicine in Europe. Data are from a survey conducted in 2014 [7] and are based on 40,185 interviews of a representative sample of the population (sample size ≥1500/country). The prevalence (% of the population/year using acupuncture, chiropractic, herbal remedies, homeopathy, osteopathy and spiritual healing) is plotted for 20 European countries

A European survey conducted in 2014 examined the use of homeopathy and other popular forms of “alternative/complementary” medicine (acupuncture, chiropractic, herbal treatment, osteopathy, spiritual healing) [7]. This survey covered 21 European countries and Israel and provided data from structured interviews with 40,185 individuals (i.e. a representative sample ≥1500/country). We retrieved the pertinent data available at http://www.europeansocialsurvey.org/data. It is evident from Fig. 1 that the prevalence of “alternative/complementary” medicine, in general, and homeopathy, in particular, varies widely in individual European countries: homeopathy is, for instance, rarely used in the Nordic countries (prevalence 1% in Denmark, Norway, Sweden and 2% in Finland), the UK and Ireland (prevalence 1%), countries from southern (prevalence 2 and 3% in Portugal and Spain, respectively) and Eastern Europe (prevalence 1–3% in the Czech Republic, Estonia, Hungary, Poland). In contrast, the use of homeopathy is highly prevalent (≥10%) in France, Switzerland, Germany and Austria. The large variations in neighboring countries with reasonably comparable socioeconomic status (e.g. the threefold difference in prevalence between The Netherlands and Germany or the >10-fold difference between Denmark and Germany) indicate that homeopathy does not cover an unmet medical need. The variation rather suggests that the use of homeopathy is shaped—at least in part—by market forces and attitudes. Here, we intend to review the plausibility of homeopathy and to explore the reasons for its continuous popularity and why homeopathy is still advocated.

## Theoretical framework of homeopathy

The principles of homeopathy were first introduced in 1796 by Samuel Hahnemann [8]; they were subsequently extended in the first edition of his book *Organon der rationellen Heilkunde/Organon of rational medicine*, which was published in 1810: 5 editions followed and the title changed repeatedly (*Organon der Heilkunst, Organon of the art of healing; Organon der Heilkunde, Organon of medicine” *is the 6th edition, which Samuel Hahnemann completed in 1842 and which is considered the definitive reference book) to account for Hahnemann’s deeper insights.

One core tenet is “similia similibus curentur” (like cures like), i.e. the principle of similarity: compounds, which can produce symptoms (at high doses), can cure a disease with similar symptoms (when administered at low doses). The groundwork for this concept of a “remedy picture” was laid down in self-experimentation. In his approach, Hahnemann was inspired by the Viennese physician Anton von Stoerck who is considered one of the fathers of pharmacology, because he had posited that drugs (in those days herbal extracts) ought to be systematically tested, first in animals, then in healthy people and finally in patients. Thus, scientific drug testing in homeopathy and in pharmacology can be traced to a common origin; however, it is readily evident that homeopathy deviated from the scientific method from the very beginning: independent replication and confirmation was not considered important. Hahnemann ingested bark of *Cinchona* (which contains quinine) and experienced malaria-like symptoms. For Hahnemann and his acolytes, this single observation sufficed as proof for the principle of similarity. Prior to and after Hahnemann’s famous self-experimentation, quinine has been ingested by many people: they did not experience any fever. Thus, Hahnemann’s observation is not reproducible. The fever, which he experienced, was likely due to an immune response. Idiosyncratic reactions to quinine have long been known [9–11]. They can be accounted for by quinidine-dependent antibodies [12, 13].

The assumptions underlying homeopathic drug testing (“proving” = homeopathic pathogenetic trial) were already questioned in the famous Nuremberg salt trial (“Nürnberger Kochsalzversuch”), a well-conducted placebo-controlled, double blind experiment [14, 15]. By 1834, homeopathy had become very popular, in particular among the affluent citizens of Nuremberg. Dismayed by the perceived irrationality of the aristocracy and moneyed bourgeoisie in his hometown, Friedrich Wilhelm von Hoven published a scathing criticism of homeopathy under the pseudonym E.F. Wahrhold. He was challenged by Johann Jacob Reuter, one of the leading homeopaths, who asserted that Wahrhold/von Hoven would experience the effects of NaCl C30, if he only tried it himself. The Society of Truth-loving Men took up the challenge and conducted a well-designed double-blind test, which was supported by George Löhner, the publisher of a newspaper: 100 new glass vials were numbered, thoroughly shuffled and divided into 2 random lots, 50 vials each were filled with melt water from snow or with NaCl C30 prepared according to the prescription of Reuter (1 grain of salt dissolved in 100 drops of melt water and serially diluted 29 times at a ratio of 1:100 in melt water; the weighing scales were new and had never been in contact with any medicinal product). The content of the vials was recorded in a list, which was sealed. The vials were distributed at random among volunteers; their name and the pertinent vial number was recorded on separate list. After 3 weeks, the volunteers (50 out of 54) reported any perceived unusual effect. Of the participants eight reported an effect, three were in the group who had ingested the pure melt water, five had ingested the NaCl C30 solution. The account was published by Löhner [14]. It was evident to the organizers of the trial that Reuter’s claim had been discredited: the vast majority of the participants had not experienced any effect. It is worth noting that this experiment was conducted and its results published, before Hahnemann completed the last edition of his book: Hahnemann chose to ignore this negative trial.

A second core tenet of homeopathy is to posit that dilution enhances the healing power of the remedy by dynamization or potentization/potentiation. In this process, the “spirit-like” power hidden in the inner essence of the remedy is released through sequential dilution and repetitive shaking. The concept is based on vitalism: living organisms were postulated to be endowed with a specific force, i.e. the *vis vitalis*, which accounted for their ability to synthesize organic compounds. This concept was falsified by Friedrich Wöhler, when he showed that it was possible to synthesize urea (1828) from inorganic precursors [16]. Of note, this breakthrough happened during Hahnemann’s lifetime but he again chose to ignore its repercussions for his theory of releasable “spirit-like” powers. Modern homeopathy invokes “water memory” to explain the actions of infinitely dilute solutions. Water does have a memory arising from the intermolecular hydrogen bond network; however, it lasts only some 50 femtoseconds [17]. Even when water molecules are confined, the hydrogen bonds rearrange with time constants in the range of 1.3 picoseconds [18]. Last but not least, homeopathy employs dilutions of compounds (e.g. “calcium carbonicum Hahnemanni” from oyster shells; “arsenicum album”, arsenic trioxide), which are present as low-level contaminants in the diluent water (or other excipients). It is intrinsically illogical that potentization can confer any specific action to the added compounds: they are present in the diluent anyway and they cannot be further diluted. Nevertheless, highly diluted solutes, i.e. NaCl and LiCl diluted below Avogado’s number, have been reported to affect the thermoluminescence of frozen, irradiated water (H_2_O and D_2_O) [19]: this study is highly popular to justify the mechanistic basis of homeopathy. It is, however, not clear how artificially created (i.e. irradiation-induced) electron holes in solid water relate to biological processes. The reported differences in thermoluminescence are most likely due to different levels of contaminants. This explanation is substantiated by thermoluminescence recordings of a study, which was designed to reproduce the original findings of Rey: the most conspicuous finding of van Wijk et al. [20] is the huge variability in photon counts; there was no statistically significant difference between heavy water (D_2_O) subjected to succussion in the absence and presence of (infinitely diluted) LiCl. The citation records indicate that the publication of Rey [19] is substantially more popular than that of van Wijk et al. [20]: according to the Scopus data base they have received 169 and 21 total citations, respectively. This suggests that there is a bias against the negative findings of Wijk et al. [20].

Homeopathy has also been invoking a hodgepodge of non-Euclidean mathematics, quantum mechanics, the theory of relativity, Schrödinger’s cat etc. for the past 70 years to account for its special approach [21–24]. Advances in quantum mechanics have been translated into outlandish theories. Particle entanglement, for instance, serves as a theoretical background for the “weak quantum theory”, which posits a double entanglement: the original homeopathic drug is entangled with its dilution and the individual symptoms of a patient are entangled with the general symptoms of a “remedy picture”, which account for the principle of potentization/potentiation and for the simile principle, respectively [23, 24]. An alternative is to postulate a triad, patient-practitioner remedy (PPR) entanglement [24, 25]. The approach and the verbiage are reminiscent of Lacan’s attempt to establish the scientific foundation of psychoanalysis in Riemann space, set theory and torus mathematics [26, 27]. More importantly, the entanglement serves as a convenient immunization strategy: in a randomized controlled trial, the entanglement is interrogated by observation; hence it must collapse. Accordingly, the homeopathic therapeutic effect is lost; thus by definition, homeopathy cannot be investigated by the gold standard of evidence-based-medicine, the double-blind placebo-controlled randomized trial. Any effect, which can be nevertheless observed, suggests that entanglement was very strong to enable the detection of a residual signal in the trial [25].

## Homeopathy and the scientific approach

It is evident that—from the very beginning—Hahnemann and his acolytes were immune to scientific evidence, which questioned their dogmatic system. In one of his major inquiries, *The Logic of Scientific Discovery*/*Logik der Forschung*, Sir Karl Popper argued that the scientific method relied on reproducibility and falsifiability [28]. Popper also acknowledged the importance of theories but emphasized that theories must be challenged, because only this leads to their improvement and thus ultimately to scientific progress [29]. From the perspective of scientific approach, homeopathy has four problems: (i) the assumptions underlying its theory lack plausibility and they violate fundamental laws of chemistry and physics, i.e. the law of mass action and the second law of thermodynamics. (ii) The theory is being immunized against scrutiny and falsification. (iii) Homeopathy has no explanatory power. (iv) Last but not least, homeopathic remedies are ineffective, if examined in adequately controlled clinical trials.

### Plausibility

The principle of similarity is a dogma, which must be believed; there is neither rational nor empirical support to justify this assumption. Human physiology is understood in considerable detail. The same is true for the pathophysiology of many diseases. There is little rationale to support the assumption that diseases can be cured by interventions, which provoke the very same disease: it is, for instance, not logical to posit that diabetes mellitus can be cured by raising glucose levels or by inducing insulin resistance. The only situation where the assumption “like cures like” is valid, is immunization by vaccines; vaccination, however, is typically rejected by practitioners of homeopathy.

Hahnemann equated symptoms and diseases, hence removing the symptoms is equivalent to healing the disease (§6 and §7 of the *Organon*). It is obvious that this assumption is fundamentally flawed. The repertory of homeopathic drugs is based on a collection of observed/perceived symptoms, which are elicited by a given drug in volunteers. As outlined above, from the very beginning, homeopathic pathogenetic trials were not reproducible. More than 200 years after the seminal self-experiment with quinidine, the repertory was still judged to rely on poorly conducted trials [30]. More recent studies are also unconvincing: the effects of ozone C30 (i.e. diluted 10^60^-fold) was most frequently equated to *Haliaeetus leucocephalus sanguinaria, *i.e. a remedy prepared from blood of the American bald eagle [32]. It is worthwhile to quote the explanation of the authors verbatim:

“The HPT (= homeopathic pathogenetic trial) databases of Ozone and *Haliaeetus leucocephalus sanguinaria* share the theme of ‘upward’ or floating sensations, such as feeling light and free, feelings of elation and euphoria and dreams of being high up in the mountains” [31].

It is obvious that there has to be more than a pinch of mysticism to accept the fact that ingestion of blood of an eagle causes these feelings. It is also questionable, whether these feelings are relevant to the treatment of any disease. Finally, it is not logical that ozone should be uplifting like an eagle: ozone is heavier than air and therefore sinks. The homeopathic literature abounds in statements such as these. They may be poetic but they are meaningless from a scientific perspective.

During the 1930s, when homeopathy enjoyed political support by the Nazi regime in Germany for ideological reasons (see above), the Imperial Ministry of Health (*Reichsgesundheitsamt*) encouraged homeopathic pathogenetic trials. These were to be carried out in a blinded and placebo-controlled fashion. The publication of the results was apparently suppressed. Fritz Donner, a German physician and homeopath, however, provided a frank account on both the trials and the dangerous machinations in clinical medicine, where patients with ulcers, pernicious anemia, gonorrhea, hyperthyroidism etc. were treated with homeopathic remedies. Proponents of homeopaths were most prone to report amazing symptoms, when challenged with placebos in the homeopathic pathogenetic trials, and to ignore the lack of therapeutic effects in their patients ([32–36] and the report can also be accessed at https://www.kwakzalverij.nl/behandelwijzen/homeopathie/der-donner-bericht/). It is worth noting that the publication of this report was actively suppressed by the Association of German Homeopaths (Deutsche Zentralverein homöopathischer Ärzte).

The law of mass action governs all chemical reactions and hence also the action of drugs: drugs bind to targets and this binding reaction elicits a biological action. Metaphorically, one can choose to refer to this binding reaction as a transfer of information, as proponents of homeopathy like to do. Nevertheless, Avogadro’s number imposes a lower limit for chemical reactions. While Avogadro’s number was not known to Hahnemann, current homeopathy cannot ignore this problem as it cannot ignore the impurities, which are present in water or any other solvent. The vast majority of water molecules, which are ingested have been present for millennia. Fortunately, when ingested, these water molecules do not bear any memory of their past exposures. It defies logical thinking to posit that homeopathic water (or any other solvent) differs from any other water or solvent of equal purity. Homeopaths credit the vigorous shaking for imparting information from the solute to the solvent. The energy, which is provided by shaking and thus presumed to create ordered water molecules (or quantal entanglement), is trivial relative to entropy: water (and other solvent) molecules move rapidly and stochastically by Brownian motion and hydrogen bonds rearrange on the femtosecond to picosecond scale [17, 18]. Thus, the second law of thermodynamics precludes any conservation of order/information. A theory, which violates these fundamental laws of nature, requires substantially larger proof than resorting to quoting Hamlet in an pseudo-erudite fashion (*There are more things in heaven and earth, Horatio, than are dreamt of in your philosophy*).

### Immunization against falsifiability

The literature on homeopathy is rife with immunization strategies: because of the unique nature of homeopathy, its essence cannot be captured by a randomized clinical trial. This is a priori not true. Randomized trials can obviously be designed to address essentially all questions in clinical medicine including those raised by homeopathy: a patient’s symptoms can be catalogued by a homeopath and the appropriate medication selected based on the resulting diagnosis. The patient can then be randomly assigned to the appropriate homeopathic remedy or to a placebo. When adequately powered (i.e. with a sufficiently large sample of patients), the beneficial effect of the homeopathic treatment ought to be evident. Because this has not been the case, patient-practitioner remedy (PPR) entanglement (see above) has been invoked [25]. Additional immunization strategies can also be found: in a trial, where placebo was—surprisingly—superior to the homeopathic remedy in treating pain associated with rheumatoid arthritis, the authors shifted the focus from the lack of efficacy to raising the issue of cost-effectiveness by stating [37]: “Instead of trying to disentangle ‘genuine’ effects of homeopathy from the placebo response, we suggest that a more directly relevant research question is whether it is cost-effective to complement conventional therapy in patients requesting homeopathy. It seems more important to define, if homeopathists can genuinely control patients’ symptoms and less relevant to have concerns about whether this is due to a ‘genuine’ effect or to influencing the placebo response”.

Homeopathic theory claims that the homeopathic remedy may initially lead to an aggravation of symptoms, which is not always seen and which is followed by a slow, but steady recovery. Aggravation serves as a convenient immunization strategy, which precludes any falsification of the treatment hypothesis: its occurrence proves the efficacy of the homeopathic remedy, but its absence is also acceptable; however, when examined in published placebo-controlled trials, aggravations are as likely to occur in patients receiving placebo as in those who were administered the homeopathic remedy [38]. In fact, aggravation and slow but steady recovery is the natural course of many diseases. In clinical trials conducted according to the principles of evidence-based medicine any aggravation must be recorded as an adverse event regardless of whether it can be linked to the treatment intervention or not.

Proponents eschew questions on reproducibility. The individualized approach to the patient and the remedy a priori provides a convenient excuse to neglect reproducibility; however, homeopathic research has also ventured into experimental studies. One of the most prominent examples is the claim that progressive dilutions of antibodies directed against IgE enhance their capacity to degranulate basophil leucocytes in human blood [39]. The statistical flaws of this study were exposed in a highly debated on-site visit [40]. Most importantly, an independent study failed to reproduce the original findings: regardless of whether it was succussed or not, anti-IgE diluted below 1: 100 did not cause any appreciable degranulation of basophils [41]. It is evident that the later publication did not receive as much media coverage as the original paper by Benveniste et al. [39].

### Lack of explanatory power

Theories, which make testable predictions, can be falsified; they can be abandoned or improved. It is evident that this drives the progress in scientific medicine: the theory of the four humours (imbalance in/dyscrasia of αἷμα/blood, χολή/yellow bile, φλέγμα/mucus and µέλαινα χολή/black bile as an explanation of disease) and the resulting dogmas of Galen of Pergamo were received, unquestioned/unquestionable knowledge for some 1500 years; however, this dogmatic view was discredited by William Harvey, when he described the circulation of blood based on experimental evidence [42]. In the past 350+ years, progress in scientific medicine has been amazing. In §1 and §8 of the *Organon*, Hahnemann ridiculed hypothesis-driven research and the approach of correlating clinical symptoms of diseases with pathological changes; however, it is remarkable that, in the past two centuries, homeopathy did not make any contribution to our current understanding of human physiology or pathophysiology: there is not a single disease entity, which can be explained by homeopathy. This is not surprising, because homeopathy does not make any testable predictions.

It has to be acknowledged, though, that homeopathy can be credited for spurring the development of blinded, placebo-controlled trials [43], of randomization and double-blinding and meticulous documentation thereof [14, 15], but this was largely an unintended consequence. Similarly, Hahnemann’s criticism of the remedies, which were administered in his day, was justified; there was little evidence to support their use, many interventions were not only ineffective but also harmful. Hence, homeopathy can also be credited for stimulating the scientific approach to pharmacotherapy [44].

### Lack of efficacy in clinical trials

Proponents of homeopathy brush aside arguments about lack of plausibility, reproducibility, falsifiability and explanatory power by stating that “whoever heals, is right”; however, when designed with relevant primary and secondary outcomes and adequately powered, homeopathic trials fail to provide any evidence for a therapeutic action, which goes beyond that seen in the placebo group [37, 45]. In fact, it appears that it is the homeopathic consultation, i.e. the interaction with the physician, rather than the homeopathic remedy, which provides a beneficial effect [46]. There are obviously numerous homeopathy trials, which report beneficial effects. These suffer from various methodological flaws. It has to be stressed that conventional scientific medicine is not immune to flawed studies. Flaws and bias can be exposed by studying individual studies, but they can also be revealed by meta-analytic approaches: Shang et al. carried out a meta-analysis of 110 homoeopathy trials and 110 matched conventional-medicine trials [47]. Matching was done for disorders and outcome measures. Many more conventional medicine than homeopathic trials have been deposited in the databases. Hence, the authors selected the matched conventional medicine trials by a randomized approach. The effect size, which was reported in each individual study, was converted into an odds ratio (i.e., the probability that the verum was superior to placebo) to allow for intertrial comparison. This exercise revealed the expected inverse correlation between study size (i.e., size of the patient population) and the normalized effect size (i.e., the odds ratio in favour of the verum); in other words: large studies reported smaller beneficial effects. This is a well-known finding, which has been seen in many meta-analyses. It is consistent with regression to the mean; however, the interesting finding of Shang et al. was the fact that in homeopathic trials the mean regressed to an odds ratio of 0.88 with a 95% confidence interval of 0.65–1.19; this is not statistically different from 1 (an odds ratio of 1 indicates that there is no difference between verum and placebo). In contrast, for the matched conventional medicines trials, the odds ratio was 0.58 with a 95% confidence interval of 0.39–0.85 (and thus significantly different from 1 indicating that the conventional medicine had an effect, which was superior to placebo). Based on this meta-analysis, the authors concluded that homeopathic trials fail to show any effect, which goes beyond that provided by placebo treatment.

The study of Shang et al. [47] published by a leading medical journal and a leading team of experts in meta-analysis, was obviously anathema to the homeopathic community and therefore heavily criticized (see e.g. [48]. and the rebuttal of the criticism [49]). An accompanying editorial in *The Lancet *pronounced the end of homeopathy, argued against “further investment in research to perpetuate the homoeopathy versus allopathy debate” and recommended that physicians be honest to their patients “about homoeopathy’s lack of benefit” [50]; however, the continuous popularity of homeopathy and the homeopathic industry still form an effective coalition to support lobbying for relaxed regulations of homeopathic remedies and for resource allocations, i.e. payments by sickness funds in various countries. These pressures have also resulted in enhanced scrutiny. Two large scale efforts, by the Australian National Health and Medical Research Council (NHMRC) in 2015 [51] and by the European Academies’ Science Advisory Council (EASAC, an association formed by the national science academies of the EU Member States) [52], were conducted to review the claims that homeopathic treatments are efficacious. Both panels concluded that none of the many studies, which had been published provided any evidence for an effect that went beyond that of a placebo.

The Australian NHMRC considered systematic reviews, published between 1997 and 2013, which compared homeopathy to placebo or other treatments. A total of 57 systematic reviews including 176 individual studies were identified, which matched the (predefined) search criteria of the panel. In addition, information was submitted to the NHRMC panel by homeopathic interest groups and the public. The NHMRC panel of researchers assessed the quality of each individual study and found that homeopathic treatment was either not better than placebo, or the studies showing superiority of homeopathy were of poor quality. Considering the body of evidence, the NHMRC panel concluded that “*… there are no health conditions for which there is reliable evidence that homeopathy is effective*” [51]. Similarly, it is worthwhile to cite the recommendations made by the European Academies’ Science Advisory Council (EASAC) [52]:

1. There should be consistent regulatory requirements to demonstrate efficacy, safety and quality of all products for human and veterinary medicine, to be based on verifiable and objective evidence, commensurate with the nature of the claims being made. In the absence of this evidence, a product should be neither approvable nor registrable by national regulatory agencies for the designation of a medicinal product.
2. Evidence-based public health systems should not reimburse homeopathic products and practices unless they are demonstrated to be efficacious and safe by rigorous testing.
3. The composition of homeopathic remedies should be labelled in a similar way to other health products available: that is, there should be an accurate, clear and simple description of the ingredients and their amounts present in the formulation.
4. Advertising and marketing of homeopathic products and services must conform to established standards of accuracy and clarity. Promotional claims for efficacy, safety and quality should not be made without demonstrable and reproducible evidence.

It will be interesting to see if these recommendations will have repercussions for regulatory authorities. The US Federal Drug Administration (FDA) has recently signalled that homeopathic drugs will come under closer scrutiny [53].

## Why people use homeopathy

**Fig. 2:**
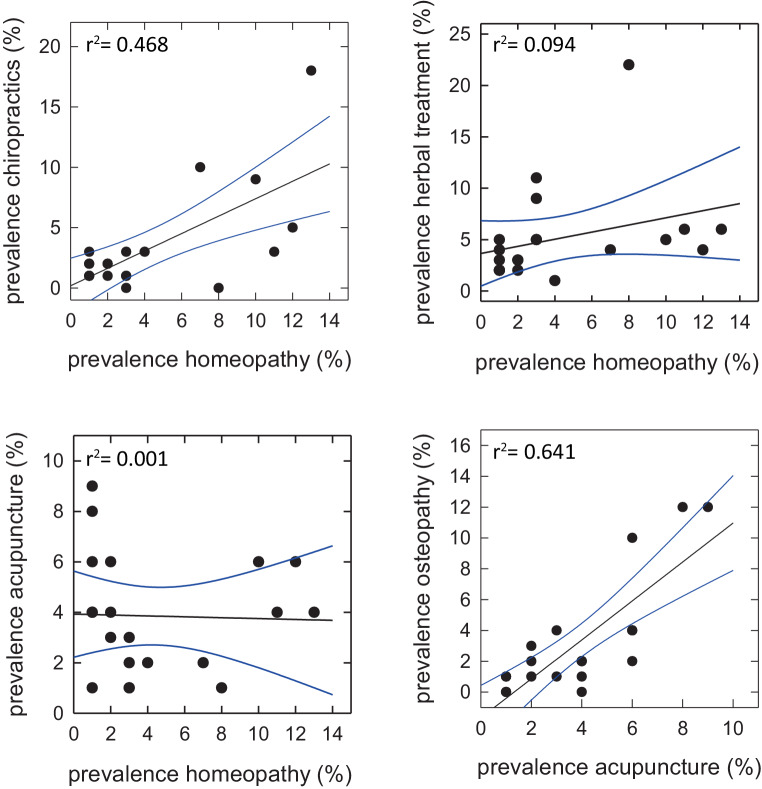
Correlation in the prevalence of different forms of “complementary/alternative” medicine in Europe. Each symbol represents the prevalence in the individual European countries shown in Fig. 1. Statistically significant correlations are only seen between the prevalence of homeopathy and of chiropractic and between the prevalence of acupuncture and of osteopathy. The blue lines indicate the 95% confidence interval

**Fig. 3:**
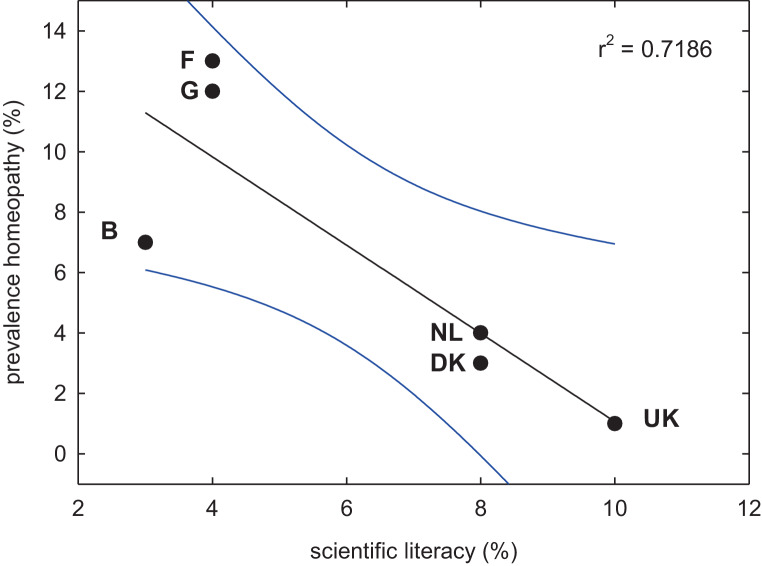
Correlation between scientific literacy and the prevalence of homeopathy in six European countries of comparable socioeconomic status. The percentage of the population with high scientific literacy according to Eurobarometer 1992 [58] was used as a measure of scientific literacy in the individual countries (*B* Belgium, *DK* Denmark, *F* France, *G* Germany, *NL* The Netherlands, *UK* The United Kingdom). The blue lines indicate the 95% confidence interval

A global survey indicated that the general public (adult of all ages) in various countries was as likely to resort to homeopathy as to acupuncture, herbal medicine and osteopathy [54]. We noted that the data from the European survey indicated wide variations in the prevalence of homeopathy in European countries (Fig. 1). We used the available data to examine if there was any correlation between the popularity of homeopathy and of other forms of “alternative/complementary” medicine. After testing all possible pairs, we found only two statistically significant correlations, i.e. a correlation between the prevalence of homeopathy and chiropractic (left hand upper panel in Fig. 2) and between the prevalence of acupuncture and osteopathy (right hand lower panel in Fig. 2). People, who resort to herbal medicine, presumably believe in the healing powers of nature. Interestingly, the prevalence of homeopathy and of herbal medicine are not linked (right hand upper panel in Fig. 2). Perhaps, less surprisingly, there is also no link between the prevalence of homeopathy and acupuncture (left hand lower panel in Fig. 2). The fact that there are few correlations is also consistent with an earlier survey: it was noted some 25 years ago that in the UK, patients who resorted to different forms of “alternative/complementary” medicine, differed with respect to their motives and to the extent to which they mistrusted orthodox (conventional) medicine [55]. Taken together, this reinforces our view that the popularity of “alternative/complementary” medicine is shaped by factors other than unmet medical needs. As outlined above, the assumptions underlying homeopathy are scientifically implausible. Scientific literacy was examined by the Eurobarometer 1992 in the 12 then member states of the European Union (European Community). We selected six countries of then comparable socioeconomic status (Belgium, Denmark, France, Germany, The Netherlands and The UK), for which data on scientific literacy of the general population were available [56]. The use of homeopathy has been stable over the past 20 years (1991–2011) [54]. Thus, assuming a little change in the prevalence of homeopathy up to 2014 and stable scientific literacy of the general population, we examined the conjecture that there was a link between scientific literacy and prevalence of homeopathy: Fig. 3 shows that for these six countries, which are in close vicinity to each other, there is indeed a significant inverse correlation between scientific literacy and prevalence of homeopathy.

It has already noted (see above Nuremberg salt trial, [15]) that from its very beginning homeopathy was popular among the upper classes. This was not only true for Germany but also for Britain [57], where royalty and the aristocracy have been supportive up to the very present with Prince Charles becoming a patron of the Faculty of Homeopathy in June 2019. It is likely that the access to homeopathy is limited by financial means. In fact, in spite of his humble beginnings, when Hahnemann died in Paris in 1843 he was a millionaire [57]. Hence, it is instructive to examine the use of homeopathy in Switzerland, where homeopathy is covered by the public healthcare system; accordingly, the use of homeopathy is less likely to be determined by socioeconomic status. Nevertheless, based on a Swiss survey, it transpires that the typical user of homeopathy is young, female, affluent and has attained a higher education level [58]. It is puzzling that educated upper class people should be so receptive to homeopathy in spite of the scientific evidence. Charles Percy Snow’s famous observations on the “two cultures” [59]: in many—if not all—Western countries a person is considered highly educated, if she or he is knowledgeable in literature and the arts, but ignorant in the most fundamental scientific principle. Snow used the example that most people, who considered themselves highly educated, failed to explain the second law of thermodynamics. Incidentally, Snow’s very example is also of relevance to homeopathy, because a precondition to accept the theoretical framework of homeopathy is to ignore the second law of thermodynamics (see above). Individuals of high social rank are overconfident and consistently overestimate their competence [60]. We propose that both socially acceptable scientific illiteracy and overconfidence account—at least in part—for the phenomenon that individuals of high social rank espouse homeopathy.

A recurrent observation in structured interviews is the finding that patients are attracted to homeopathy, because they are dissatisfied by one or several aspects of conventional medicine [61, 62]. It is evident that the homeopathic consultation caters to needs, which are not met by conventional medicine and this is per se beneficial [46]. Last but not least, the use of homeopathy is also socially reinforced [61, 62].

In developed (European) welfare states, patients have access to all proven treatments: by law, they must not be denied any treatment, which is justified by the scientific evidence. It is to a certain extent understandable that people, who are otherwise healthy, resort to homeopathy for minor ailments. It is less clear, why patients with cancer resort to regimens of “alternative/complementary” medicine including homeopathy, although the scientific evidence does not support their use [63]. The recurrent theme is that patients want to do as much as possible to cure their disease [64–66]. Hearsay is important in promoting the use of “alternative/complementary” medicine among cancer patients [64, 65] and there is again strong evidence for social reinforcement: patients attending support groups are more likely to use “alternative/complementary” medicine [66].

## Conclusion

It has repeatedly been pointed out that there no such thing as “alternative/complementary medicine”; there is only one medicine, i.e. medicine, which has been tested and which has been shown to work [67]. The rest is ethically unjustifiable. Medical practice presumes that the responsible patient makes an informed decision. This requires a level of health education and scientific literacy, which is obviously not met. Institutions that train medical students cannot condone the use of homeopathy if they are serious about evidence-based medicine. Homeopathic remedies must be subjected to the same regulatory requirements as other drugs for market approval. At the very least they must be labelled according to the recommendations of the European Academies’ Science Advisory Council.

## References

[1] Stehlin I (1996). Homeopathy: real medicine or empty promises?. FDA Consum.

[2] Relton C, Cooper K, Viksveen P, Fibert P, Thomas K (2017). Prevalence of homeopathy use by the general population worldwide: a systematic review. Homeopathy.

[3] Ernt E, Kaptchuk TJ (1996). Homeopathy revisited. Arch Intern Med.

[4] Kenny MG (2002). A darker shade of green: medical botany, homeopathy, and cultural politics in interwar Germany. Soc Hist Med.

[5] Ernst E (2001). „Neue Deutsche Heilkunde“: complementary/alternative medicine in the Third Reich. Complement Ther Med.

[6] Fisher P, Ward A (1994). Complementary medicine in Europe. BMJ.

[7] Eikemo TA, Bambra C, Huijts T, Fitzgerald R (2017). The first pan-European sociological health inequalities survey of the general population: the European social survey rotating module on the social determinants of health. Eur Sociol Rev.

[8] Hahnemann S. Versuch über einen neues Prinzip zur Auffindung der Heilkräfte der Arzneisubstanzen, nebst einigen Blicken auf die bisherigen (“Essay on a new principle for ascertaining the curative powers of drugs, together with perspectives on the existing principles”). C.W. Hufelands Journal der practischen Arzneykunde und der Wundarzneykunst. 1796; 2: 391–439 & 465–561.

[9] Griffith SF (1877). Peculiar effects from exhibition of quinine. Ohio Med Rec.

[10] Gibb W (1888). A case of remittent fever in a child aged two years and nine months, with an idiosyncrasy towards quinine. Glasgow Med J.

[11] Grenier F (1898). A case of idiosyncrasy of intolerance of quinine. Ind Med Gaz.

[12] Gottschall JL, Neahring B, McFarland JG, Wu GG, Weitekamp LA, Aster RH (1994). Quinine-induced immune thrombocytopenia with hemolytic uremic syndrome: clinical and serological findings in nine patients and review of literature. Am J Hematol.

[13] Hou M, Horney E, Stockelberg D, Jacobsson S, Kutti J, Wadenvik H (1997). Multiple quinine-dependent antibodies in a patient with episodic thrombocytopenia, neutropenia, lymphocytopenia, and granulomatous hepatitis. Blood.

[14] Loehner G, A Society of Tuth-Loving Men (1835). Die homoeopathischen Kochsalzversuche zu Nürnberg [The homeopathic salt trials in Nuremberg].

[15] Stolberg M (2006). Inventing the randomized double-blind trial: the Nuremberg salt test of 1835. J R Soc Med.

[16] Wöhler F (1828). Über künstliche Bildung des Harnstoffs (On the artificial formation of urea). Ann Phys Chem.

[17] Cowan ML, Bruner BD, Huse N, Dwyer JR, Chugh B, Nibbering ET, Elsaesser T, Miller RJ (2005). Ultrafast memory loss and energy redistribution in the hydrogen bond network of liquid H_2_O. Nature.

[18] Gilijamse JJ, Lock AJ, Bakker HJ (2005). Dynamics of confined water molecules. Proc Natl Acad Sci U S A.

[19] Rey LR (2003). Thermoluminescence of ultra-high dilutions of lithium chloride and sodium chloride. Phys A.

[20] van Wijk R, Bosman S, van Wijk EP (2006). Thermoluminescence in ultra-high dilution research. J Altern Complement Med.

[21] Roberston T (1949). Homoeopathy and recent advances in science and semantics. Br Homoeopath J.

[22] Walach H (2003). Entanglement model of homeopathy as an example of generalized entanglement predicted by weak quantum theory. Forsch Komplementarmed Klass Naturheilkd.

[23] Weingärtner O (2007). The nature of the active ingredient in ultramolecular dilutions. Homeopathy.

[24] Weingärtner O (2005). The homeopathic mechanism from the viewpoint of a quantum mechanical paradoxon. J Altern Complement Med.

[25] Milgrom L (2002). Patient–Practitioner–Remedy (PPR) entanglement: Part 1. A qualitative, non-local metaphor for homeopathy based on quantum theory. Homeopathy.

[26] Dawkins R (1998). Postmodernism disrobed. Nature.

[27] Sokal A, Bricmont J (1998). Fashionable nonsense: postmodern intellectuals’ abuse of science.

[28] Popper KR (1959). The logic of scientific discovery.

[29] Popper KR (1963). Conjectures and Refutations: The growth of scientific knowledge.

[30] Dantas F, Fisher P, Walach H, Wieland F, Rastogi DP, Teixeira H, Koster D, Jansen JP, Eizayaga J, Alvarez ME, Marim M, Belon P, Weckx LL (2007). A systematic review of the quality of homeopathic pathogenetic trials published from 1945 to 1995. Homeopathy.

[31] Sherr J, Quirk T, Tournier AL (2014). Do homeopathic pathogenetic trials generate recognisable and reproducible symptom pictures?: results from a pilot pathogenetic trial of Ozone 30c. Homeopathy.

[32] Donner F (1969). Observation faites lors des vérifications relatives aux méthodes de l’homéopathie [Observations made during the examination of homeopathic methods]. Cah Biother.

[33] Donner F (1995). Bemerkungen zu der Überprüfung der Homöopathie durch das Reichsgesundheitsamt 1936–39. Teil I. Die Vorbereitungsphase [Comments on the examination by the Reichsgesundheitsamt of homeopathy 1936–39. Part I. The preparatory phase. Perfusion.

[34] Donner F (1995). Bemerkungen zu der Überprüfung der Homöopathie durch das Reichsgesundheitsamt 1936–39. Teil II. Das Konzept Comments on the examination by the Reichsgesundheitsamt of homeopathy 1936–39. Part I. The concept. Perfusion.

[35] Donner F (1995). Bemerkungen zu der Überprüfung der Homöopathie durch das Reichsgesundheitsamt 1936–39. Teil III. Probleme. Comments on the examination by the Reichsgesundheitsamt of homeopathy 1936–39. Part III. Probl Perfus.

[36] Donner F (1995). Bemerkungen zu der Überprüfung der Homöopathie durch das Reichsgesundheitsamt 1936–39. Teil IV. Experimente und Ergebnisse. Comments on the examination by the Reichsgesundheitsamt of homeopathy 1936–39. Part IV. Experiments and results. Perfusion.

[37] Fisher P, Scott DL (2001). A randomized controlled trial of homeopathy in rheumatoid arthritis. Rheumatology.

[38] Grabia S, Ernst E (2003). Homoeopathic aggravations: a systematic review of randomised, placebo-controlled clinical trials. Homeopathy.

[39] Davenas E, Beauvais F, Amara J, Oberbaum M, Robinzon B, Miadonna A, Tedeschi A, Pomeranz B, Fortner P, Belon P, Sainte-Laudy J, Poitevin B, Benveniste J (1988). Human basophil degranulation triggered by very dilute antiserum against IgE. Nature.

[40] Maddox J, Randi J, Stewart WW (1988). “High-dilution” experiments a delusion. Nature.

[41] Hirst SJ, Hayes NA, Burridge J, Pearce FL, Foreman JC (1993). Human basophil degranulation is not triggered by very dilute antiserum against human IgE. Nature.

[42] Harvey W. Exercitatio Anatomica de Motu Cordis et Sanguinis in Animalibus (Anatomical account of the motion of the heart and blood in animals). Frankfurt; 1628.

[43] Dean ME (2006). ‘An innocent deception’: placebo controls in the St Petersburg homeopathy trial, 1829–1830. J R Soc Med.

[44] Forbes J (1846). Homoeopathy, allopathy and “young physic”. Br Foreign Med Rev.

[45] Paris A, Gonnet N, Chaussard C, Belon P, Rocourt F, Saragaglia D, Cracowski JL (2007). Effect of homeopathy on analgesic intake following knee ligament reconstruction. Br J Clin Pharmacol.

[46] Brien S, Lachance L, Prescott P, McDermott C, Lewith G (2011). Homeopathy has clinical benefits in rheumatoid arthritis patients that are attributable to the consultation process but not the homeopathic remedy: a randomized controlled clinical trial. Rheumatology.

[47] Shang A, Huwiler-Müntener K, Nartey L, Jüni P, Dörig S, Sterne JA, Pewsner D, Egger M (2005). Are the clinical effects of homoeopathy placebo effects? Comparative study of placebo-controlled trials of homoeopathy and allopathy. Lancet.

[48] Rutten AL, Stolper CF (2008). The 2005 meta-analysis of homeopathy: the importance of post-publication data. Homeopathy.

[49] Wilson P (2009). Analysis of a re-analysis of a meta-analysis: in defence of Shang et al. Homeopathy.

[50] The Lancet (2005). The end of homoeopathy. The Lancet.

[51] National Health and Medical Research Council (2015). NHMRC information paper: evidence on the effectiveness of homeopathy for treating health conditions.

[52] European Academies’ Science Advisory Council. Homeopathic products and practices: assessing the evidence and ensuring consistency in regulating medical claims in the EU. 2017. https://easac.eu/publications/details/homeopathic-products-and-practices/. Accessed: 27 Oct 2019

[53] Podolsky SH, Kesselheim AS (2016). Regulating homeopathic products—a century of dilute interest. N Engl J Med.

[54] Cooper KL, Harris PE, Relton C, Thomas KJ (2013). Prevalence of visits to five types of complementary and alternative medicine practitioners by the general population: a systematic review. Complement Ther Clin Pract.

[55] Furnham A, Vincent C, Wood R (1995). The health beliefs and behaviors of three groups of complementary medicine and a general practice group of patients. J Altern Complement Med.

[56] Miller JD (1998). The measurement of civic scientific literacy. Public Understand Sci.

[57] Loudon I (2006). A brief history of homeopathy. J R Soc Med.

[58] Klein SD, Torchetti L, Frei-Erb M, Wolf U (2015). Usage of complementary medicine in Switzerland: results of the Swiss health survey 2012 and development since 2007. PLoS ONE.

[59] Belmi P, Neale MA, Reiff D, Ulfe R (2020). The social advantage of miscalibrated individuals: the relationship between social class and overconfidence and its implications for class-based inequality. J Pers Soc Psychol.

[60] Snow CP (1959). The two cultures.

[61] Avina RL, Schneiderman LJ (1978). Why patients choose homeopathy. West J Med.

[62] Gunther M (1999). The homeopathic patient: comparative results of homeopathic and conventional GP patient interviews. Med Ges Gesch.

[63] Smith PJ, Clavarino A, Long J, Steadman KJ (2014). Why do some cancer patients receiving chemotherapy choose to take complementary and alternative medicines and what are the risks?. Asia Pac J Clin Oncol.

[64] Humpel N, Jones SC (2006). Gaining insight into the what, why and where of complementary and alternative medicine use by cancer patients and survivors. Eur J Cancer Care.

[65] Morant R, Jungi WF, Koehli C, Senn HJ (1991). Warum benützen Tumorpatienten Alternativmedizin? (Why do cancer patients use alternative medicine?). Schweiz Med Wochenschr.

[66] Boon H, Stewart M, Kennard MA, Gray R, Sawka C, Brown JB, McWilliam C, Gavin A, Baron RA, Aaron D, Haines-Kamka T (2000). Use of complementary/alternative medicine by breast cancer survivors in Ontario: prevalence and perceptions. J Clin Oncol.

[67] Angell M, Kassirer JP (1998). Alternative medicine—the risks of untested and unregulated remedies. N Engl J Med.

